# Taste Neurons Consist of Both a Large TrkB-Receptor-Dependent and a Small TrkB-Receptor-Independent Subpopulation

**DOI:** 10.1371/journal.pone.0083460

**Published:** 2013-12-27

**Authors:** Da Fei, Robin F. Krimm

**Affiliations:** Department of Anatomical Sciences and Neurobiology, University of Louisville, School of Medicine, Louisville, Kentucky, United States of America; Baylor College of Medicine, United States of America

## Abstract

Brain-derived neurotrophic factor (BDNF) and neurotrophin-4 (NT-4) are two neurotrophins that play distinct roles in geniculate (taste) neuron survival, target innervation, and taste bud formation. These two neurotrophins both activate the tropomyosin-related kinase B (TrkB) receptor and the pan-neurotrophin receptor p75. Although the roles of these neurotrophins have been well studied, the degree to which BDNF and NT-4 act via TrkB to regulate taste development *in vivo* remains unclear. In this study, we compared taste development in *TrkB^−/−^* and *Bdnf^−/−^/Ntf4^−/−^* mice to determine if these deficits were similar. If so, this would indicate that the functions of both BDNF and NT-4 can be accounted for by TrkB-signaling. We found that *TrkB^−/−^* and *Bdnf^−/−^/Ntf4^−/−^* mice lose a similar number of geniculate neurons by E13.5, which indicates that both BDNF and NT-4 act primarily via TrkB to regulate geniculate neuron survival. Surprisingly, the few geniculate neurons that remain in *TrkB^−/−^* mice are more successful at innervating the tongue and taste buds compared with those neurons that remain in *Bdnf^−/−^/Ntf4^−/−^* mice. The remaining neurons in *TrkB^−/−^* mice support a significant number of taste buds. In addition, these remaining neurons do not express the TrkB receptor, which indicates that either BDNF or NT-4 must act via additional receptors to influence tongue innervation and/or targeting.

## Introduction

The cell bodies of taste neurons are located in the geniculate ganglion. During development, taste neurons innervate specific regions of the gustatory epithelium with a precise number of neurons. The development of the geniculate ganglion and the formation of peripheral connections are highly regulated by two neurotrophins, brain-derived neurotrophic factor (BDNF) and Neurotrophin-4 (NT-4). *Bdnf^−/−^* and *Ntf4^−/−^* mice each lose approximately 50% of their geniculate neurons by birth [1], [2]. However, NT-4 regulates the survival of geniculate neurons at an earlier stage of development compared with BDNF via a different mechanism [3]. BDNF in the tongue epithelium also directs gustatory axons to their correct targets, while NT-4 does not [4], [5]. *Bdnf^−/−^* mice, which have a deficit in targeting, have a more severe loss of innervation to taste buds than *Ntf4^−/−^* mice [3], [6]–[8].

BDNF and NT-4 function via the TrkB and p75 receptors [9], [10]. Mice that lack TrkB lose more nodose-petrosal ganglion neurons than BDNF or NT-4 mutants [1], which indicates that both BDNF and NT-4 may act via TrkB to influence neuron development. Consistently, both *Bdnf^−/−^/Ntf4^−/−^* and *TrkB^−/−^* mice lose approximately 95% of their geniculate neurons [2], [11]. The maintenance of taste buds and fungiform papillae requires innervation by birth, and a lack of innervation results in taste bud loss in both *Bdnf* ^−/−^ and *Ntf4* ^−/−^ mice at birth [7], [12]. Surprisingly, a substantial number of fungiform papillae and taste buds remain at birth in *TrkB^−/−^* mice [11], while *Bdnf^−/−^/Ntf4^−/−^* mice exhibit a substantial loss of fungiform papillae and taste buds at birth [13]. The reasons for the differences in the developing taste system in *Bdnf^−/−^/Ntf4^−/−^* and *TrkB^−/−^* are unclear. Furthermore, the results are difficult to compare because the two studies used different quantification methods. Therefore, two open questions remain: 1) How much do BDNF and NT-4 act via TrkB to regulate geniculate neuron survival and taste bud innervation during development?; and 2) Why do taste buds develop in *TrkB^−/−^* mice despite the substantial loss of taste neurons?

To address these questions, we directly compared the development of the gustatory system in *Bdnf^−/−^/Ntf4^−/−^* and *TrkB^−/−^* mice. These data provide direct evidence that BDNF and NT-4 act primarily via TrkB to regulate the survival of taste neurons *in vivo*. However, a small subpopulation of these geniculate neurons does not require or express TrkB, and these neurons can innervate and support the development of a substantial number of taste buds.

## Materials and Methods

### Animals

Heterozygous *TrkB^+/−^* (stock no. 002544), *Bdnf^+/−^* (stock no. 002266), and *Ntf4^+/−^* (stock no. 002497) mice were acquired from Jackson Laboratories (Bar Harbor, Maine, USA). *TrkB^tauEGFP^* mice were a generous gift from Dr. David Ginty [14]. *TrkB^−/−^* embryos were obtained by breeding heterozygous mice with a target mutation of the *TrkB* gene. *TrkB^tauEGFP/−^* embryos were obtained by breeding *TrkB^tauEGFP^* mice with *TrkB^+/−^* mice. *Bdnf^−/−^/Ntf4^−/−^* mice were obtained by breeding *Bdnf^+/−^/Ntf4^−/−^* mice. Animals were genotyped using polymerase chain reaction. Embryonic mice were identified from female mice that were bred and examined for plugs the following morning. The day that a plug was positively identified was designated as embryonic day 0.5. *The care, handling, and use of all animals followed the guidelines of the U.S. Public Health Service Policy on Humane Care and Use of Laboratory Animals and the NIH Guide for the Care and Use of Laboratory Animals. All procedures used in this study were approved by the University of Louisville Institutional Animal Care and Use Committee (IACUC#10074).*

### Quantification of Geniculate Ganglion Volume and Neuron Number

#### Staining

Embryos aged E11.5 (*TrkB^−/−^* n = 3, *Bdnf^−/−^/Ntf4^−/−^* n = 3, and wild-type n = 3), E12.5 (*TrkB^−/−^* n = 3, *Bdnf^−/−^/Ntf4^−/−^* n = 3, and wild-type n = 3), and E13.5 (*TrkB^−/−^* n = 3, *Bdnf^−/−^/Ntf4^−/−^* n = 3, and wild-type n = 3) were transcardially perfused with ice-cold 4% phosphate-buffered paraformaldehyde (PFA). Following perfusion, embryos were post-fixed overnight in 4% PFA. Following fixation, embryo heads were dissected, moved to 70% ethanol, and processed for paraffin embedding. Geniculate ganglion neurons were visualized by class III β-tubulin (TUJ-1) antibody as previously described [15]. Briefly, serial sections (5 µm) of paraffin-embedded embryos were collected on SuperFrost Plus slides (Fisher Scientific, Pittsburgh, PA, USA). Paraffin was removed by immersion in Citrisolv overnight. Following rehydration and endogenous peroxidase blocking, slides were treated for antigen retrieval in a citrate buffer (0.1 M citric acid, 0.1 M sodium citrate, dH_2_O; pH 6). Sections were washed in phosphate-buffered saline (PBS), blocked for 1 h in a blocking solution (PBS, 5% goat serum, 0.25% Triton X-100), and incubated overnight in mouse anti-β-III tubulin antibody (1∶500, Covance, Princeton, NJ, USA; catalog #MMS-435P) in a blocking solution. On the following day, sections were washed and incubated for 1.5 h in biotinylated anti-mouse secondary antibody (1∶250, Vector Laboratories, Burlingame, CA, USA; #BA-2000) in a blocking solution, and visualized with an ABC diaminobenzidine reaction kit (Vector Laboratories, Burlingame, CA, USA; #PK-6200).

#### Quantification

The area of the geniculate ganglion was measured in each section and multiplied by the section thickness (5 µm) to compute the volume of the geniculate ganglion within a single section; these volumes were added to compute the total volume of the entire ganglion. The TUJ-1 antibody was used to identify and count neuronal profiles in sections in which the nucleus was visible to quantify the number of geniculate neurons. Neuronal profiles were counted in six representative sections per ganglion. The geniculate ganglion volume in each of these six sections was also measured. The total number of neuronal profiles of the entire ganglion was estimated as the product of the number of profiles per volume of the counted section multiplied by the total volume of the entire ganglion. The total number of neurons per ganglion was estimated by multiplying the number of total neuron profiles by a correction factor to compensate for the presence of a nucleus in multiple sections [16]. The correction factor was calculated from the formula: N = n×[T/(T×D)], where N is the estimated total number of neurons, n is the number of nuclear profiles, T is the measured section thickness, and D is the average diameter of the nuclei [15].

### Quantification of Fungiform Papilla Number using a Scanning Electron Microscope (SEM)

Mice (*TrkB^−/−^* n = 3, *Bdnf^−/−^/Ntf4^−/−^* n = 3, and wild-type n = 5) were anesthetized on the day of birth and transcardially perfused with ice-cold 4% PFA. The tongues were dissected and post-fixed in 1% aqueous OsO4 for 2.0 to 2.5 h, washed in buffer, and successively dehydrated in a graded series of ethanol and hexamethyldisilazane (HMDS). The tongues were housed in a desiccator overnight to evaporate the HMDS. On the following day, the tongues were mounted onto stubs, sputter-coated with gold, and examined by SEM (Phillips 505). Digital SEM images were captured at 130×magnification to distinguish the fungiform and filiform papillae. Individual fungiform papillae were imaged at 1770×magnification.

### Quantification of Taste Bud Number

Mice (*TrkB^−/−^* n = 5, *Bdnf^−/−^/Ntf4^−/−^* n = 5, and wild-type n = 4) were anesthetized on the day of birth and transcardially perfused with ice-cold 4% PFA. The front of the tongue, which contains the fungiform field, was separated and post-fixed in 4% PFA for 2 h. The tongues were placed overnight in 30% sucrose as a cryoprotectant. On the following day, the tongues were embedded in OCT (Sakura Finetek USA, Torrance, CA, USA, #4583). Serial sagittal sections of the tongue (25 µm) were collected onto SuperFrost Plus slides (Fisher Scientific, Pittsburgh, PA, USA). Sections were heat-dried overnight, rehydrated, placed into citrate buffer (pH 6.0), heated for 15 min in a boiling water bath, and incubated for 10 min at room temperature (RT) to retrieve antigens. The slides were washed in PBS and incubated overnight in 1∶50 rat anti-TROMA1 antibodies (Developmental Studies Hybridoma Bank, Iowa City, IA, USA) in PBS. On the following day, the slides were rinsed in PBS (3 times for 5 min each) and incubated in anti-rat Alexa 555 secondary antibodies (1∶500, Molecular Probes, Eugene, OR, USA) for 2 h. After washing in PBS (3 times for 5 min each), the slides were dehydrated, cleared in Citrisolv, and cover-slipped using a DPX mounting medium (Fluka, St. Louis, MO, USA). The sections were examined in order, and the taste buds were followed across sections to ensure that each taste bud was counted only once.

### Quantification of the Amount of P2X3 Labeled Innervation within a Taste Bud

The same procedures as described above were used to identify the taste buds in another set of mice (*TrkB^−/−^* n = 4, *Bdnf^−/−^/Ntf4^−/−^* n = 4, and wild-type n = 4). Following the procedures for antigen retrieval, the tongues were incubated overnight with both rat anti-Troma1 antibody and rabbit anti-P2X3 antibody (1∶500, Millipore, Billerica, MA, USA, #AB5895) as primary antibodies to label the taste fibers. Secondary anti-rat Alexa 488 (green) and anti-rabbit Alexa 555 (red) antibodies (1∶500, Molecular Probes, Eugene, OR, USA) were also used to visualize the taste buds and taste fibers, respectively.

Confocal stacks of optical sections with a Z step of 0.5 were imaged for 3 to 5 taste buds from every mouse within each genotype and analyzed by the ImageJ software (Version 1.43, 22 April 2010, http://rsbweb.nih.gov/ij/). The area occupied by the taste bud in each image section was measured, and areas were summed and multiplied by the section thickness (0.5 µm) to calculate the taste bud volume. The area occupied by P2X3-positive staining within the outlined taste bud was also measured in each optical section. These areas were summed and multiplied by the section thickness (0.5 µm) to measure the volume of innervation within the taste bud. The percentage of the taste bud that was occupied by the innervation was determined by dividing the volume of the P2X3 label by the volume of the Troma1 label.

### Geniculate Ganglion Labeling using DiI

Embryos at ages E14.5, E15.5, E16.5, and E18.5 were anesthetized and transcardially perfused in ice-cold 4% PFA. The tongues were post-fixed in 4% PFA overnight. On the following day, DiI labeling was performed as described previously [17]. Embryos were incubated at 37°C for 2 to 8 weeks depending on the age of the embryo. The tongue was then dissected, examined, and photographed using a fluorescent dissecting microscope (Leica MZFL) equipped with a camera (QImaging CE). Images were collected from the tongues of *TrkB^−/−^, Bdnf^−/−^/Ntf4^−/−^,* and wild-type mice at the following ages: E14.5 (*TrkB^−/−^* n = 4, *Bdnf^−/−^/Ntf4^−/−^* n = 3, and wild-type n = 3), E15.5 (*TrkB^−/−^* n = 4, *Bdnf^−/−^/Ntf4^−/−^* n = 3, and wild-type n = 5), E16.5 (*TrkB^−/−^* n = 6, *Bdnf^−/−^/Ntf4^−/−^* n = 4, and wild-type n = 3), and E18.5 (*TrkB^−/−^* n = 4, *Bdnf^−/−^/Ntf4^−/−^* n = 3, and wild-type n = 5).

### Quantification of the Innervation to the Lingual Epithelium

Embryos at E16.5 (*TrkB^−/−^* n = 3, *Bdnf^−/−^/Ntf4^−/−^* n = 3, and wild-type n = 3) were anesthetized and transcardially perfused with ice-cold 4% PFA. The front of the tongue, which contains the fungiform field, was separated and post-fixed for 2 h. The tongues were placed overnight in 30% sucrose and embedded in OCT (Sakura Finetek USA, Torrance, CA, USA, #4583) the following day. Serial sagittal sections of the tongue (25 µm) were collected onto SuperFrost Plus slides (Fisher Scientific, Pittsburgh, PA, USA). Sections were heat-dried overnight, rehydrated, placed into citrate buffer (pH 6.0), heated for 15 min in a boiling water bath, and incubated for 10 min at RT to retrieve antigens. The slides were washed in PBS and incubated overnight in mouse anti-2H3 antibody (1∶100, Developmental Studies Hybridoma Bank, Iowa City, IA, USA) and rabbit anti-P2X3 antibody (1∶500, Millipore, Billerica, MA, USA, #AB5895) in PBS. The slides were rinsed in PBS (3 times for 5 min each) and incubated in anti-rabbit Alexa 488 and anti-mice Alexa 555 secondary antibodies (1∶500, Molecular Probes, Eugene, OR, USA) for 2 h. After washing in PBS (3 times for 5 min each), the slides were dehydrated, cleared in Citrisolv, and cover-slipped using a DPX mounting medium (Fluka, St. Louis, MO, USA). The sections were examined in order. Each instance in which the nerve fibers invaded the epithelium was quantified. Each location was examined in serial sections to ensure that each invading fiber bundle was counted only once.

### Quantification of TrkB-GFP Expression in the Geniculate Ganglion

Embryos at E13.5 (*TrkB^ tauEGFP/−^* n = 3 and wild-type n = 3) were anesthetized and transcardially perfused in ice-cold 4% PFA. The head was dissected and post-fixed overnight in 4% PFA. The head was then placed during the following overnight period in 30% sucrose as a cryoprotectant. On the following day, the tissue was embedded in OCT (Finetek USA, Torrance, CA, USA, #4583) and the heads were sectioned at 25 µm and collected onto SuperFrost Plus slides (Fisher Scientific, Pittsburgh, PA, USA). The slides were allowed to dry at 40°C for 1 h. The slides were rinsed 3 times for 5 min each in PBST (PBS with 2.5% triton), blocked in 5% normal serum in PBST for 1 h, and incubated overnight at RT in chicken anti-GFP (1∶1000, Invitrogen, Grand Island, NY, USA #A11122) and rabbit anti-P2X3 (1∶500, Millipore, Billerica, MA, USA, #AB5895) antibodies in a blocking solution. On the following day, the slides were rinsed in PBST (3 times for 5 min each) and incubated in anti-chicken Alexa 488 and anti-rabbit Alexa 555 (1∶500, Molecular Probes, Eugene, OR, USA) secondary antibodies for 1 h. After washing in PBST (4×5 min), the slides were mounted with fluoromount-G (SouthernBiotech, Birmingham, Alabama, USA #0100-01). The number of GFP- and/or P2X3-positive neurons were counted. The ratio of the number of neurons that expressed both TrkB and P2X3 compared with the number of neurons that expressed P2X3 only was calculated.

### Data Analysis

The total neuron number and total volumes were compared between genotypes on embryonic days E11.5, E12.5 and E13.5 using a two-way analysis of variance (ANOVA, IBM SPSS version 20). The fungiform papillae number and area, the taste bud number and volume, and the taste bud innervation data were compared using a one-way ANOVA. The alpha levels were set at p<0.05 for all statistical comparisons. The data are reported as the mean ± S.E.M.

## Results

### BDNF and NT-4 Both Act Primarily via TrkB to Support Geniculate Ganglion Neuron Survival during Embryonic Development

BDNF and NT-4 have been shown to regulate gustatory neuron development at different embryonic stages. *Ntf4^−/−^* mice start to lose geniculate neurons at E11.5, but *Bdnf^−/−^* mice do not start to lose geniculate neurons until E13.5 [3], [15]. Both *Bdnf^−/−^* and *Ntf4^−/−^* mice lose approximately half of their geniculate neurons by birth. To understand whether the regulation of geniculate neuron loss by these two neurotrophins acts via the same receptor, TrkB, we quantified the number of geniculate neurons in wild-type, *Bdnf^−/−^/Ntf4^−/−^,* and *TrkB^−/−^* mice from E11.5 to E13.5. We reasoned that if TrkB mediates the effects of these two ligands, then *Bdnf^−/−^/Ntf4^−/−^* and *TrkB^−/−^* mice would lose a similar number of geniculate neurons over the same timeframe.

**Figure 1:**
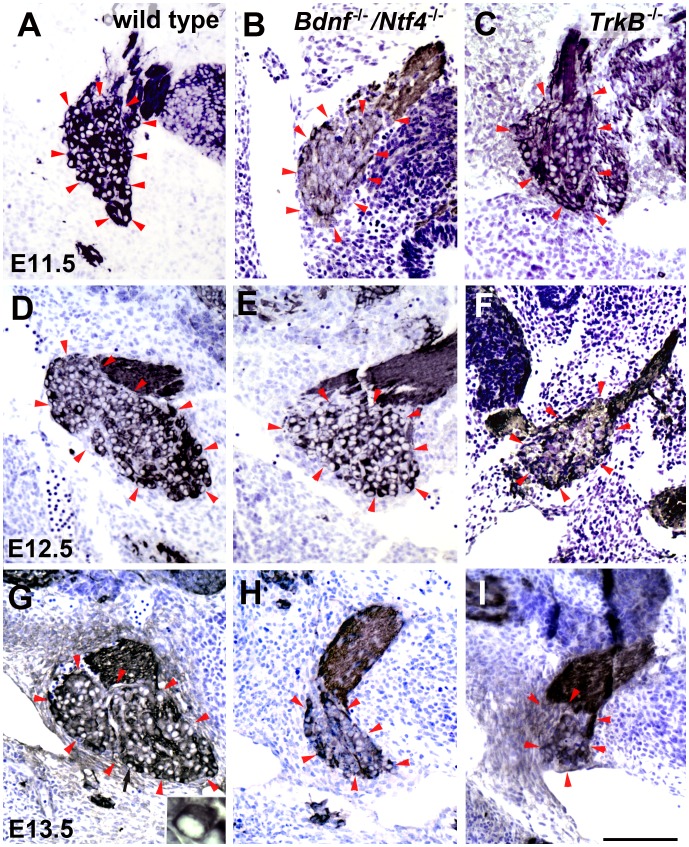
TUJ-1 labeled geniculate ganglia from *Bdnf^−/−^/Ntf4^−/−^* and *TrkB^−/−^* mice decrease in size compared with wild-type mice between E11.5 and E13.5. Paraffin-embedded sections were stained with TUJ-1 antibody. This antibody is against β-tubulin and labels the cytoplasm of neurons because of the large number of microtubules present. The sections were counterstained with nissl stain which also stains the cytoplasm of neuron cell bodies. At E11.5, the geniculate ganglion was similar in size in wild-type (A), *Bdnf^−/−^/Ntf4^−/−^* (B), and *TrkB^−/−^* mice (C). Red arrow heads define the borders of the geniculate ganglion. At E12.5, the size of the geniculate ganglion was reduced in *Bdnf^−/−^/Ntf4^−/−^* (E) and *TrkB^−/−^* (F) mice compared with wild-type mice (D). The size of the geniculate ganglion appeared larger at E13.5 compared with E11.5 in wild-type mice (G compared with A). However, the size of the geniculate ganglion continued to decrease in *Bdnf^−/−^/Ntf4^−/−^* (H) and *TrkB^−/−^* mice (I) at E13.5. The inset in panel G shows the geniculate ganglion at a higher magnification to illustrate a cell (indicated by black arrow) that was positively labeled for the neuronal marker TUJ-1 with a dark cytoplasm and a clear nucleus. The scale bar in I = 100 µm and applies to A–I. The inset is 5X larger than the original image.

**Figure 2:**
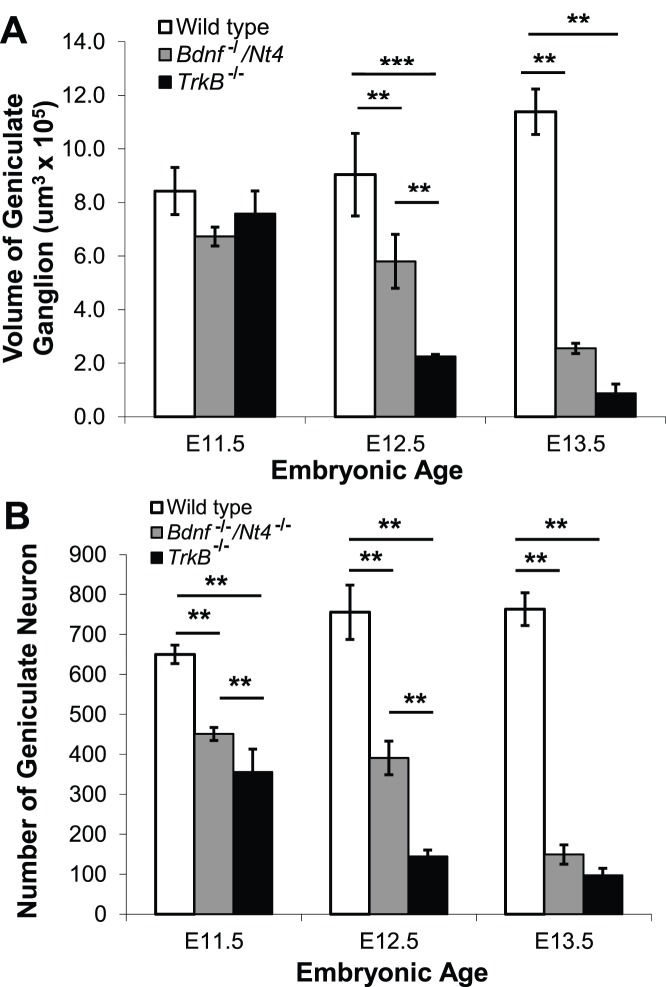
The volume and number of geniculate ganglion neurons decrease in *Bdnf^−/−^/Ntf4^−/−^* and *TrkB^−/−^* mice between E11.5 and E13.5. (A) At E11.5, there was no difference between the geniculate ganglion volumes in wild-type, *Bdnf^−/−^/Ntf4^−/−^,* and *TrkB^−/−^* mice. At E12.5, the geniculate ganglion volume was reduced by 36% and 76% in *Bdnf^−/−^/Ntf4^−/−^* and *TrkB^−/−^* mice, respectively, compared with wild-type mice. The geniculate ganglion volume was significantly smaller in the *TrkB^−/−^* compared with the *Bdnf^−/−^/Ntf4^−/−^* mice. There was a significant increase in the geniculate ganglion volume in wild-type mice at E13.5 compared with E11.5. There were significant reductions in the geniculate ganglion volumes of *Bdnf^−/−^/Ntf4^−/−^* and *TrkB^−/−^* mice at E13.5, but no significant difference between the two mutant genotypes at this stage. (B) At E11.5, the number of geniculate neurons was reduced by 31% and 45% in the *Bdnf^−/−^/Ntf4^−/−^* and *TrkB^−/−^* mice compared with the wild-type mice. At E12.5, the number of geniculate neurons was reduced by 48% and 81% in the *Bdnf^−/−^/Ntf4^−/−^* and *TrkB^−/−^* mice, respectively, compared with the wild-type mice. There were also significantly fewer neurons in the *TrkB^−/−^* mice than the *Bdnf^−/−^/Ntf4^−/−^* mice at E12.5. At E13.5, the number of geniculate neurons was reduced by 80% and 87% in *Bdnf^−/−^/Ntf4^−/−^* and *TrkB^−/−^* mice, respectively, compared with the wild-type mice. There was no significant difference in the number of neurons between the two mutant genotypes at E13.5. *p<0.05, **p<0.01, and ***p<0.001.

First, we compared geniculate ganglion volumes in wild-type, *Bdnf^−/−^/Ntf4^−/−^*_,_ and *TrkB^−/−^* mice. At E11.5, there were no differences in geniculate ganglion volume between genotypes: wild-type (8.4±0.88×10^5^ µm^3^), *Bdnf^−/−^/Ntf4^−/−^* (6.7±0.35×10^5^ µm^3^), and *TrkB^−/−^* mice (7.6±0.85×10^5^ µm^3^) (Figure 1A, B, C; Figure 2A). At E12.5, geniculate ganglion volume was reduced by 36% (p<0.01) in *Bdnf^−/−^/Ntf4^−/−^* (5.8±1.01×10^5^ µm^3^) and 76% (p<0.001) in *TrkB^−/−^* (2.2±0.08×10^5^ µm^3^) compared with wild-type mice (9.0±1.54×10^5^ µm^3^). The geniculate ganglion volume was significantly smaller in *TrkB^−/−^* compared with *Bdnf^−/−^/Ntf4^−/−^* mice (p<0.01) (Figure 1D, E, F; Figure 2A). At E13.5, the geniculate ganglion volume increased in wild-type mice (p<0.05) and continued to decrease in both *Bdnf^−/−^/Ntf4^−/−^* and *TrkB^−/−^* mice (p<0.01). There was no significant difference between the geniculate ganglion volumes in *Bdnf^−/−^/Ntf4^−/−^* and *TrkB^−/−^* mice at E13.5. These results show that geniculate ganglion volume increases in wild-type mice during early development, but is significantly reduced in *Bdnf^−/−^/Ntf4^−/−^* and *TrkB^−/−^* mice. Furthermore, the geniculate ganglion volume reduction is slightly delayed in *Bdnf^−/−^/Ntf4^−/−^* mice compared with *TrkB^−/−^* mice.

Next, we quantified the number of neurons present in the geniculate ganglion. Geniculate neurons were easy to identify because they have a clear nucleus and dark cytoplasm when stained with TUJ-1 antibody (Figure 1G). There was a slight increase (17%; p<0.01) in the number of geniculate neurons in wild-type mice from E11.5 to E13.5. However, there was a continuous neuronal loss in *Bdnf^−/−^/Ntf4^−/−^* and *TrkB^−/−^* littermates over this same embryonic period (p<0.01; Figure 2B). Specifically, at E11.5, the number of geniculate neurons in *Bdnf^−/−^/Ntf4^−/−^* (451±16) and *TrkB^−/−^* mice (355±58) was reduced by 31% (p<0.01) and 45% (p<0.01), respectively, compared with wild-type mice (650±23) (Figure 2B). There were 21% fewer geniculate neurons in *TrkB^−/−^* compared with *Bdnf^−/−^/Ntf4^−/−^* mice (p<0.01) at this age. This finding is consistent with earlier studies, which found that neuron number is a more sensitive measure of cell loss than geniculate volume during early development when neurons are small [3]. At E12.5, there was a 16% increase in the number of geniculate neurons in the wild-type mice (756±68) compared with the E11.5 littermates (p<0.01, Figure 2B). Although there was no significant change in the number of geniculate neurons in the *Bdnf^−/−^/Ntf4^−/−^* mice at E12.5 compared with E11.5, *Bdnf^−/−^/Ntf4^−/−^* mice had 48% fewer geniculate neurons compared with the wild-type mice (p<0.01), which is similar to the neuron loss that has been observed in *Ntf4^−/−^* mice [3]. However, the number of geniculate neurons in *TrkB^−/−^* mice (144±16) decreased 59% between ages E11.5 and E12.5 (p<0.01) and was reduced by 81% compared with wild-type littermates (p<0.01), which is consistent with an earlier study that showed a substantial loss of geniculate neurons in *TrkB^−/−^* mice by E12.5 [12]. There were also 64% fewer neurons in *TrkB^−/−^* compared with *Bdnf^−/−^/Ntf4^−/−^* mice at E12.5 (p<0.01). One explanation for this finding is that other neurotrophins, such as NT-3, may also act via TrkB to regulate neuronal survival during early development. At E13.5, the number of geniculate neurons was reduced by 80% and 87% in *Bdnf^−/−^/Ntf4^−/−^* (149±24; p<0.01) and *TrkB^−/−^* mice (97±18; p<0.01), respectively, compared with wild-type mice (763±41, Figure 2B). Therefore, the bulk of the ganglion has disappeared in both genotypes by E13.5. Together, these data suggest a continuous neuron loss in both BDNF/NT-4 and TrkB mutant genotypes between E11.5 and E13.5. This loss is equivalent by E13.5, which indicates that BDNF and NT-4 act primarily via TrkB to regulate neuron survival during early development.

### *TrkB^−/−^* Mice Lose the Same Number of Fungiform Papillae but have More Taste Buds than *Bdnf^−/−^/Ntf4^−/−^* Mice by Birth

**Figure 3:**
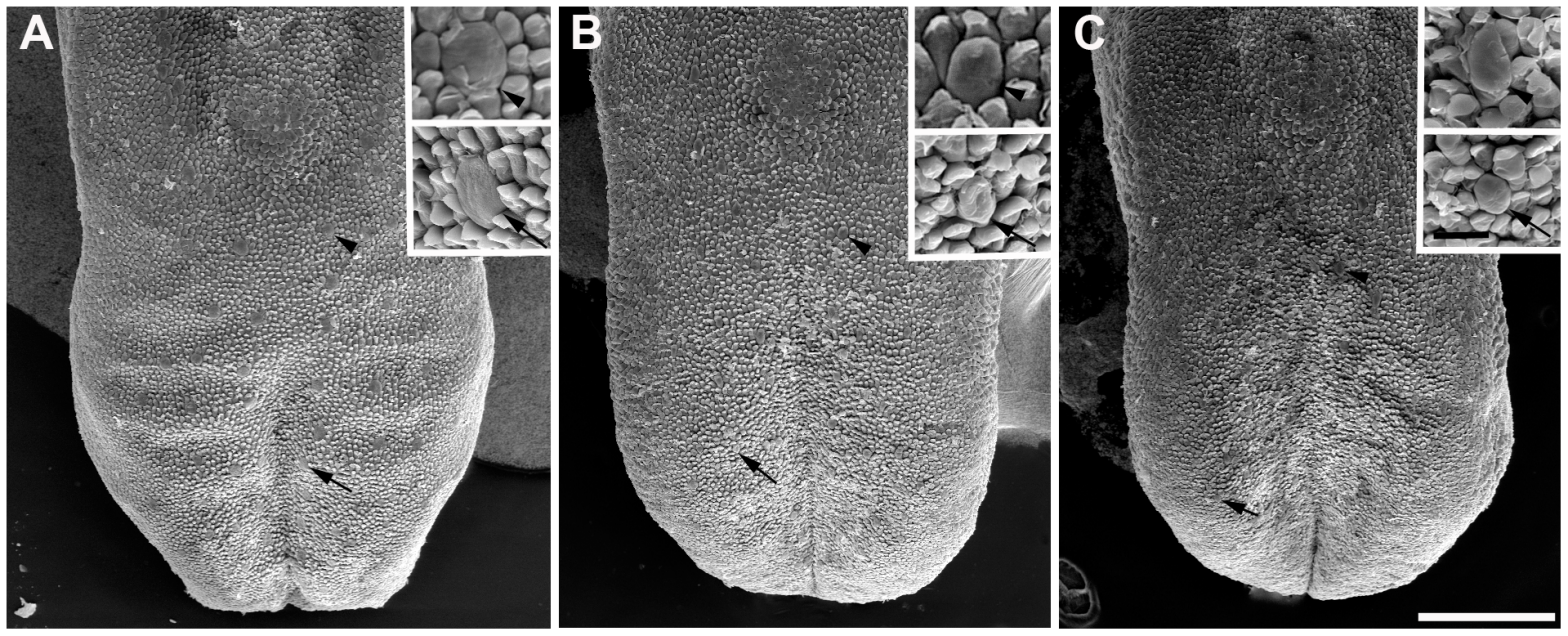
*Bdnf^−/−^/Ntf4^−/−^* and *TrkB^−/−^* mice have fewer and smaller fungiform papillae than wild-type mice at P0. The number and size of the fungiform papillae were quantified with scanning electron microscopy (SEM) images of the tongue from wild-type (A), *Bdnf^−/−^/Ntf4^−/−^* (B), and *TrkB^−/−^* mice (C). Two insets in each panel illustrate individual fungiform papillae at higher magnification from the tip (arrow) and back (arrowhead) of the tongue of each genotype. There were significantly fewer fungiform papillae at P0 in *Bdnf^−/−^/Ntf4^−/−^* (61±5) and *TrkB^−/−^* (53±2) compared with wild-type mice (84±2, p<0.01), but there was no significant difference in the number and size of the fungiform papillae between the two mutant genotypes. The scale bar in C = 300 µm and applies to A–C; the scale bar in the inset panels = 40 µm and applies to the three inset panels in A–C.

Post-natal taste buds are supported by innervation from neuronal fibers. If the chorda tympani nerve is severed during development, then the taste buds and fungiform papillae are lost [18]–[20]. Many neurotrophin knockout studies have demonstrated a loss of taste buds at birth due to the earlier loss of geniculate neurons [3], [6], [7], [12], [13], [15], [21]. However, minimal taste bud loss was observed in *TrkB* ^−/−^ mice despite a significant loss of geniculate neurons [11], but the number of taste buds was not quantified. Therefore, in this study, both the fungiform papilla and taste buds were quantified in *Bdnf^−/−^/Ntf4^−/−^* and *TrkB^−/−^* mice at day of birth (Figure 3). There was a significant reduction in the number of fungiform papillae at P0 in *Bdnf^−/−^/Ntf4^−/−^* (61±5) and *TrkB^−/−^* (53±2) compared with wild-type (84±2, p<0.01) mice, but there was no significant difference between the two mutant genotypes. There was also a reduction of 32% and 35% in the surface area of the fungiform papillae in *Bdnf^−/−^/Ntf4^−/−^* (132±6 µm^2^) and *TrkB^−/−^* (126±8 µm^2^) mice, respectively, compared with wild-type mice (195±12 µm^2^) (Figure 3). There was no difference in the size of the fungiform papillae between *Bdnf^−/−^/Ntf4^−/−^* and *TrkB^−/−^* mice.

**Figure 4:**
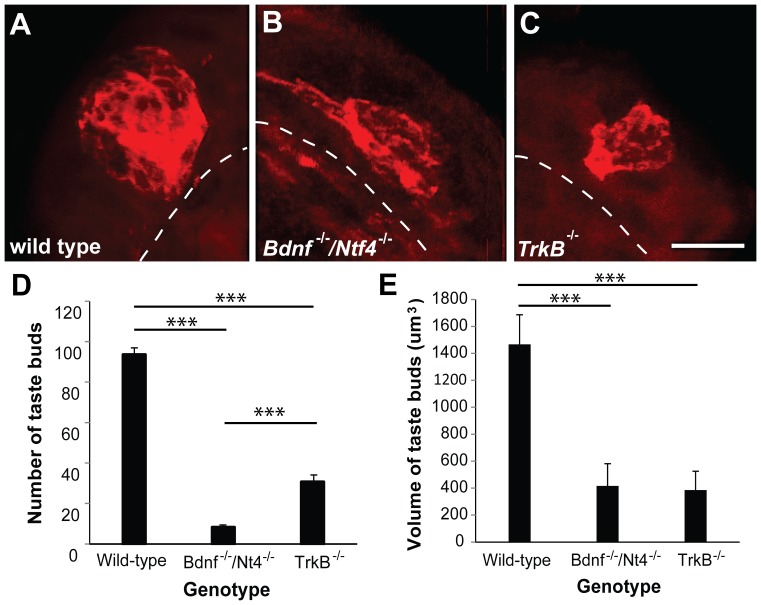
*Bdnf^−/−^/Ntf4^−/−^* mice lose more taste buds than *TrkB^−/−^* mice at P0. The taste buds were visualized by anti-Troma1 (red) staining in wild-type (A), *Bdnf^−/−^/Ntf4^−/−^* (B), and *TrkB^−/−^* mice (C). A dashed line separates the papilla epithelium from the papilla core. The number of taste buds was reduced by 87% and 67% in *Bdnf^−/−^/Ntf4^−/−^* and *TrkB^−/−^* mice, respectively; there were significantly more taste buds in the *TrkB^−/−^* mice than the *Bdnf^−/−^/Ntf4^−/−^* mice (D). The taste bud volume was smaller in the *Bdnf^−/−^/Ntf4^−/−^* and *TrkB^−/−^* mice compared with the wild-type mice; however, there was no significant difference between the two mutant genotypes (E). The scale bar in C = 10 µm and applies to A–C. **p<0.01 and ***p<0.001.

Although the number and size of fungiform papillae were reduced in *Bdnf^−/−^/Ntf4^−/−^* and *TrkB^−/−^* mice compared with wild-type mice, many fungiform papillae remained despite the severe neuron loss in these mutant genotypes. We tested the possibility that the smaller fungiform papillae lacked taste buds by examining the fungiform taste buds from *Bdnf^−/−^/Ntf4^−/−^*, *TrkB^−/−^*, and wild-type mice at P0. Taste buds were visualized using anti-Troma1 (Figure.4 A, B, C). There was a substantial loss of taste buds in both *Bdnf^−/−^/Ntf4^−/−^* (8±1) and *TrkB^−/−^* (31±3) compared with wild-type (93±3, p<0.001) mice (Figure 4D), although more taste buds were observed in *TrkB^−/−^* compared with *Bdnf^−/−^/Ntf4^−/−^* mice (p<0.001). Taste bud volume was also smaller in both mutant genotypes compared with wild-type mice (p<0.001); however, no difference in taste bud volume was observed between the mutant genotypes (Figure 4E).

### More P2X3-positive Label of Fungiform Taste Buds was Observed in *TrkB^−/−^* Compared with *Bdnf^−/−^/Ntf4^−/−^* Mice at P0

**Figure 5:**
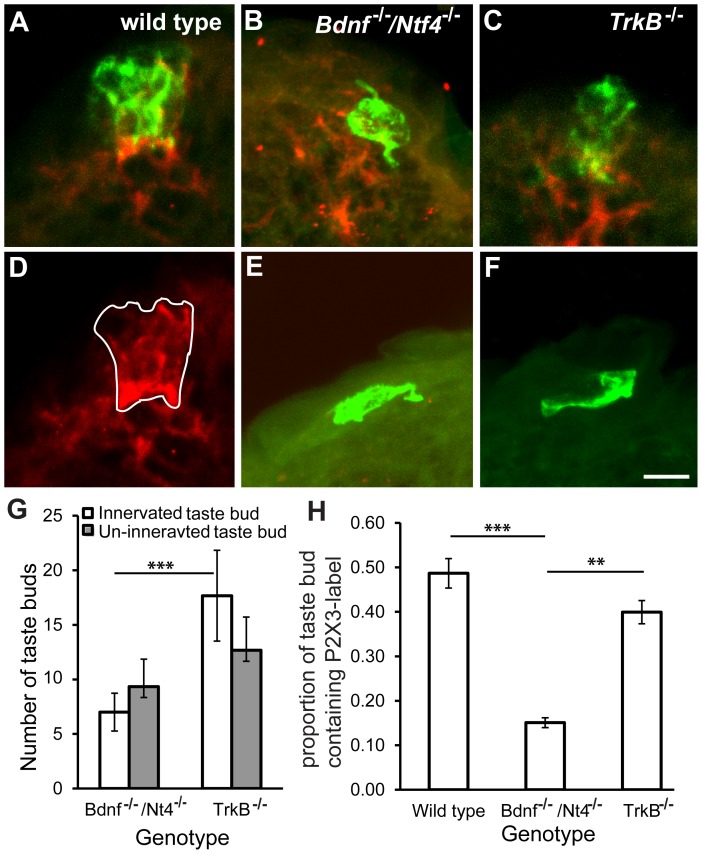
The remaining taste buds had more innervation in *TrkB^−/−^* compared with *Bdnf^−/−^/Ntf4^−/−^* mice at P0. Anti-Troma1 (green) and anti-P2X3 (red) were used to visualize the taste buds and taste innervation, respectively, in wild-type (A), *Bdnf^−/−^/Ntf4^−/−^* (B), and *TrkB^−/−^* mice (C). One optical section (D) shows the area that was occupied by the P2X3-positive nerve fibers within the taste bud. Some non-P2X3-innervated taste buds were observed in the *Bdnf^−/−^/Ntf4^−/−^* (E) and *TrkB^−/−^* mice, as defined by a cytokeratin 8-positive label (F). These taste buds appear to lack the normal taste bud morphology. (G) A comparison of the number of P2X3-innervated and P2X3-non-innervated taste buds in *Bdnf^−/−^/Ntf4^−/−^* and *TrkB^−/−^* mice indicates that there are more P2X3-innervated taste buds in *TrkB^−/−^* mice. (H) For those taste buds that were innervated, the percentage of the taste bud that is occupied by P2X3-positive nerve fibers was compared. There is more innervation with in *TrkB^−/−^* taste buds than *Bdnf^−/−^/Ntf4^−/−^*taste buds. The scale bar in F = 10 µm and applies to A–F. **p<0.01.

Taste buds are supported by gustatory innervation that must be present by birth [8], [22]–[24]. Because more taste buds were observed in the *TrkB^−/−^* compared with *Bdnf^−/−^/Ntf4^−/−^* mice at P0, we tested the possibility that the taste buds in the *TrkB^−/−^* mice had more innervation. Taste buds and taste nerves were visualized with anti-Troma1 and anti-P2X3, respectively. P2X3 is an ATP receptor that is required for gustatory nerves to respond to taste stimuli [25]. Anti-P2X3 appears to label most gustatory innervation and few trigeminal fibers [25], [26]. Taste buds that were innervated by P2X3-positive fibers (Figure 5 A, B, C) and those that were not innervated (Figure 5 E, F) were counted in *Bdnf ^−/−^/Ntf4^−/−^* and *TrkB^−/−^* mice (Figure 5G). There were significantly more taste buds innervated by P2X3-positive fibers in *TrkB^−/−^* compared with *Bdnf^−/−^/Ntf4^−/−^* mice (p<0.05). There were no differences in the number of non-P2X3-innervated taste buds in *TrkB^−/−^* and *Bdnf^−/−^/Ntf4^−/−^* mice. These results suggest that the extra taste buds that are observed in *TrkB^−/−^* compared with *Bdnf^−/−^/Ntf4^−/−^* mice are also innervated, which indicates that more taste innervation to the tongue surface exists in *TrkB^−/−^* compared with *Bdnf^−/−^/Ntf4^−/−^* mice.

Innervated taste buds also appeared to have more innervation in *TrkB^−/−^* compared with *Bdnf^−/−^/Ntf4^−/−^* mice. To measure the amount of innervation within individual taste buds, the volumes occupied by the taste bud (Troma1) and taste nerve fibers (P2X3) within each taste bud were measured for each optical section (Figure 5D). In wild-type mice, the taste bud volume was 1441±227.3 µm^3^ and the volume of P2X3-positive nerves was 678±146.7 µm^3^, which indicates that approximately 50% of the taste bud is innervated in wild-type mice at birth (Figure 5H). The proportion of the taste bud that was innervated was substantially reduced in *Bdnf^−/−^/Ntf4^−/−^* mice (p<0.001, Figure 5H): the taste bud volume was 313±37.7 µm^3^ and the volume of P2X3-positive nerves was 46±5.2 µm^3^. The proportion of the taste bud that was innervated in *TrkB^−/−^* mice (taste bud volume = 321±35 µm^3^, volume of P2X3-positive nerves = 129±9.2 µm^3^) was not significantly different from the wild-type mice, but higher than the proportion of the taste bud that was innervated in *Bdnf^−/−^/Ntf4^−/−^* mice (p<0.01) (Figure. 5H). Together, these data suggest that the remaining taste buds in *TrkB^−/−^* mice are more heavily innervated than the taste buds in *Bdnf^−/−^/Ntf4^−/−^* mice at birth.

### More Taste Fibers Reach their Targets in *TrkB^−/−^* Mice Compared with *Bdnf^−/−^/Ntf4^−/−^* Mice during Development

**Figure 6:**
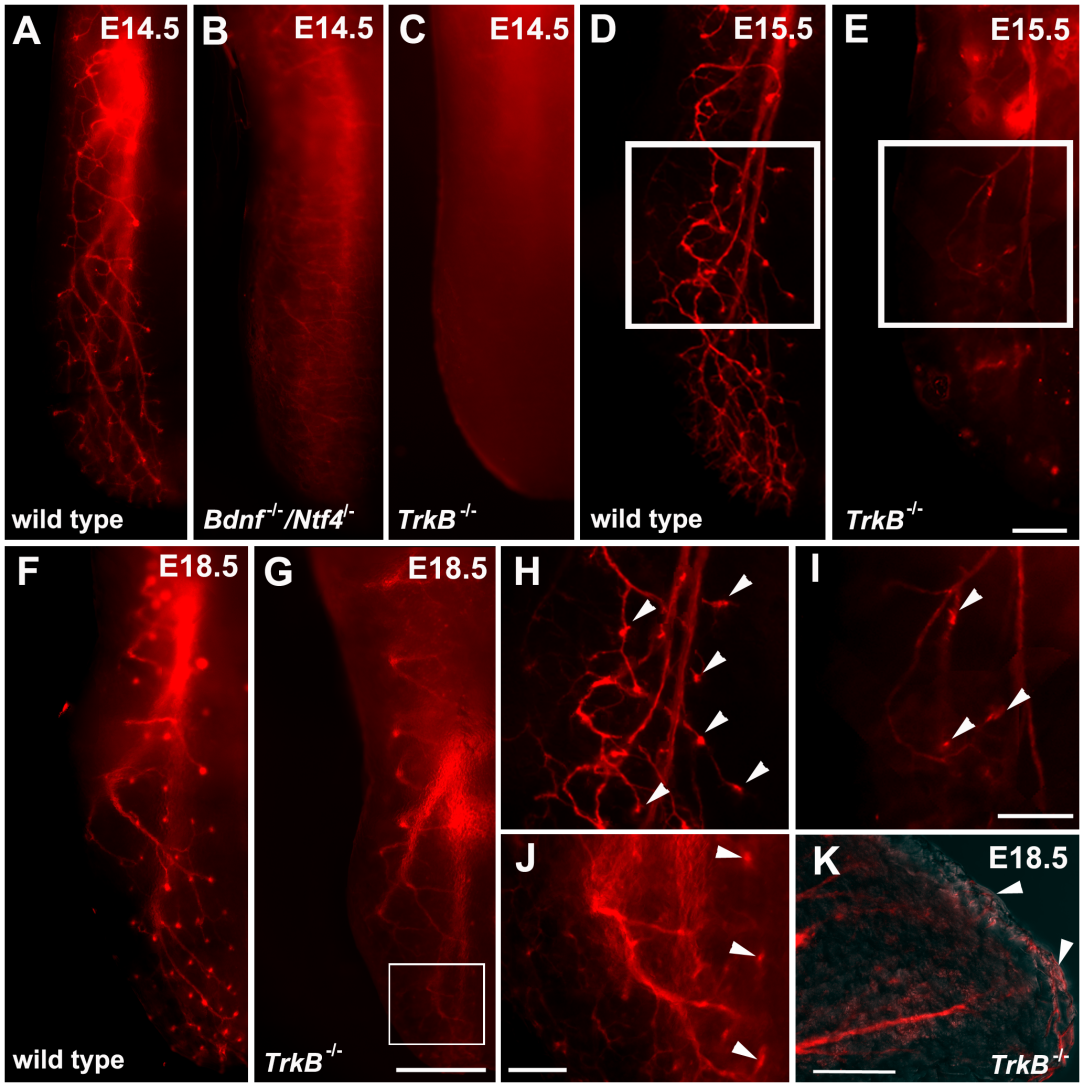
Innervation to the tongue surface remained in *TrkB^−/−^* mice. Half tongues following DiI-labeling of the geniculate ganglion in wild-type (A, D, F), *Bdnf^−/−^/Ntf4^−/−^* (B), and *TrkB^−/−^* mice (C, E, G) at E14.5, E15.5, and E18.5. A high magnification view of the tongue at E15.5 (H and I, which correspond to the boxed areas in D and E, respectively) and E18.5 (J, which corresponds to the boxed area in G) illustrates that some DiI-labeled innervation is visible to the tongue surface at E15.5 in *TrkB* ^−/−^ mice. (K) A sagittal section of the innervation to the tip of the tongue in E18.5 *TrkB^−/−^* mice also illustrates innervation reaching the tongue surface. The scale bar in E = 200 µm and applies to A–E. The scale bar in G = 500 µm and applies to F and G. The scale bar in I = 200 µm and applies to H and I. The scale bar in J = 300 µm. The scale bar in K = 500 µm.

To determine whether *TrkB^−/−^* mice have more innervation to the tongue compared with *Bdnf^−/−^/Ntf4^−/−^* mice, we labeled chorda tympani axons with the lipophilic tracer, DiI, to examine the innervation pattern in the tongue. At E14.5, chorda tympani fibers branch from the base of the tongue toward the surface in wild-type mice. We have previously shown that chorda tympani fibers defasciculate and form a structure called a “neural bud” as the fibers invade the epithelium (Figure 6A, [5], [27]) At E14.5, there were no visible fibers on the dorsal surface of the tongue in *Bdnf^−/−^/Ntf4^−/−^* or *TrkB^−/−^* mice (Figure 6B, C), which suggests that either too few chorda tympani axons remain to innervate the tongue or the growth of the axons is delayed in these mutant genotypes.

We examined the innervation patterns of the chorda tympani fibers at ages E15.5 and E18.5 to determine whether the growth of chorda tympani fibers is delayed in the *Bdnf^−/−^/Ntf4^−/−^* and *TrkB^−/−^* mice. We also examined whether more fibers reach the target in *TrkB^−/−^* compared with *Bdnf^−/−^/Ntf4^−/−^* mice. At later embryonic stages, wild-type mice had similar innervation patterns to those observed at E14.5 (Figure 6 A, D, F). At E15.5, fiber bundles branched from the base of the tongue toward the lingual surface to form a neural bud in wild-type mice (Figure 6H). However, in *Bdnf^−/−^/Ntf4^−/−^* mice, fibers did not branch to the surface of the tongue at E15.5. Furthermore, in rare cases in which innervation was observed at the epithelial surface in *Bdnf^−/−^/Ntf4^−/−^* mice, only thin wispy branches that lacked neural buds were observed. These findings are similar to previous observations in *Bdnf^−/−^/Ntf4^−/−^* mice [5]. By E18.5, there was virtually no innervation to the tongue in *Bdnf^−/−^/Ntf4^−/−^* mice (images not shown). There was clear innervation to the tongue in *TrkB^−/−^* mice at E15.5 (Figure 6 E, G), but the number of branches was fewer compared with wild-type littermates due to the loss of geniculate neurons. From E15.5 to E18.5, a few chorda tympani fibers appeared to reach the tongue surface in *TrkB^−/−^* mice (Figure 6 I, J, K arrowheads). The fiber bundles were so thin that it was difficult to determine whether the fibers invaded the epithelial surface.

**Figure 7:**
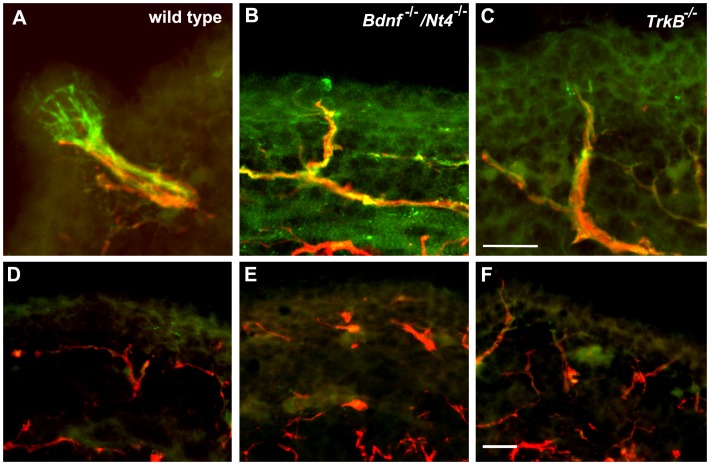
A comparison of taste nerve fibers at E16.5. Anti-P2X3 (green) and anti-neurofilament (red) antibodies were used to label taste nerve fibers in wild-type (A, D), *Bdnf^−/−^/Ntf4^−/−^* (B, E), and *TrkB^−/−^* mice (C, F). While only wild type mice had P2X3-positive neural buds innervating fungiform placodes (A), both hybrid *Bdnf* ^−/−^/*Nt4* ^−/−^ mice (B) and *TrkB* ^−/−^ mice (C) had P2X3 positive fibers penetrating the epithelium of fungiform placodes. Neurofilament-positive and P2X3-negative innervation was present in the epithelium of all three genotypes (D, E, F). The scale bar in C and F = 20 µm and applies to A–C or D–F, respectively.

To determine whether the additional innervation that was observed in the tongues of *TrkB^−/−^* compared with *Bdnf^−/−^/Ntf4^−/−^* mice also invaded the tongue epithelium, tongues at E16.5 were double-stained with anti-P2X3 (green) and anti-Neurofilament (red) (Figure 7). While many fibers in the tongue were double-labeled with these two markers, P2X3 was more effective at labeling the neural bud ending (Figure 7A). There were some thin P2X3-negative but neurofilament positive fibers in the lamina propria of all genotypes (Figure 7 D, E, F); these fibers rarely invaded the epithelium and were likely from the trigeminal nerve. Although all three genotypes had some P2X3-positive nerve fibers that invaded the epithelium, only the wild type mice had a clear P2X3-positive neural bud (Figure 7 B, C compared with Figure 7A), innervation in both knockout genotypes did not have an expanded ending. Also, the endings do not appear to differ in size between *TrkB^−/−^* and *Bdnf^−/−^/Ntf4^−/−.^* This was a bit surprising since a larger proportion of the taste bud will be occupied by P2X3 label in *TrkB^−/−^* mice compared with *Bdnf^−/−^/Ntf4^−/−^* mice by birth. This finding implies a slightly greater expansion of the neural bud between E16.5 and birth in *TrkB^−/−^* mice compared with *Bdnf^−/−^/Ntf4^−/−^* mice. However it should be noted that remaining taste buds in *TrkB* ^−/−^ mice are considerably smaller than wild type mice, consistent with the observation that both *TrkB^−/−^* and *Bdnf^−/−^/Ntf4^−/−^* have substantially smaller neural endings than wild type mice.

The number of locations at which the fibers penetrated the epithelium was quantified in serial sections. There were fewer locations where the nerve fibers penetrated the epithelium within the tongues of the *TrkB^−/−^* (21±3) and *Bdnf^−/−^/Ntf4^−/−^* mice (8±2) compared with the wild-type mice (107±6). However, there were significantly more locations with innervation in *TrkB^−/−^* compared with *Bdnf^−/−^/Ntf4^−/−^* mice despite the significant loss of innervation in both mutant genotypes, which indicates that *TrkB^−/−^* mice have more gustatory innervation than *Bdnf^−/−^/Ntf4^−/−^* mice. This greater amount of taste innervation may account for the higher number of taste buds that were observed in *TrkB^−/−^* mice.

### The Geniculate Neurons that Remain in *TrkB^−/−^* Mice do not Express TrkB

**Figure 8:**
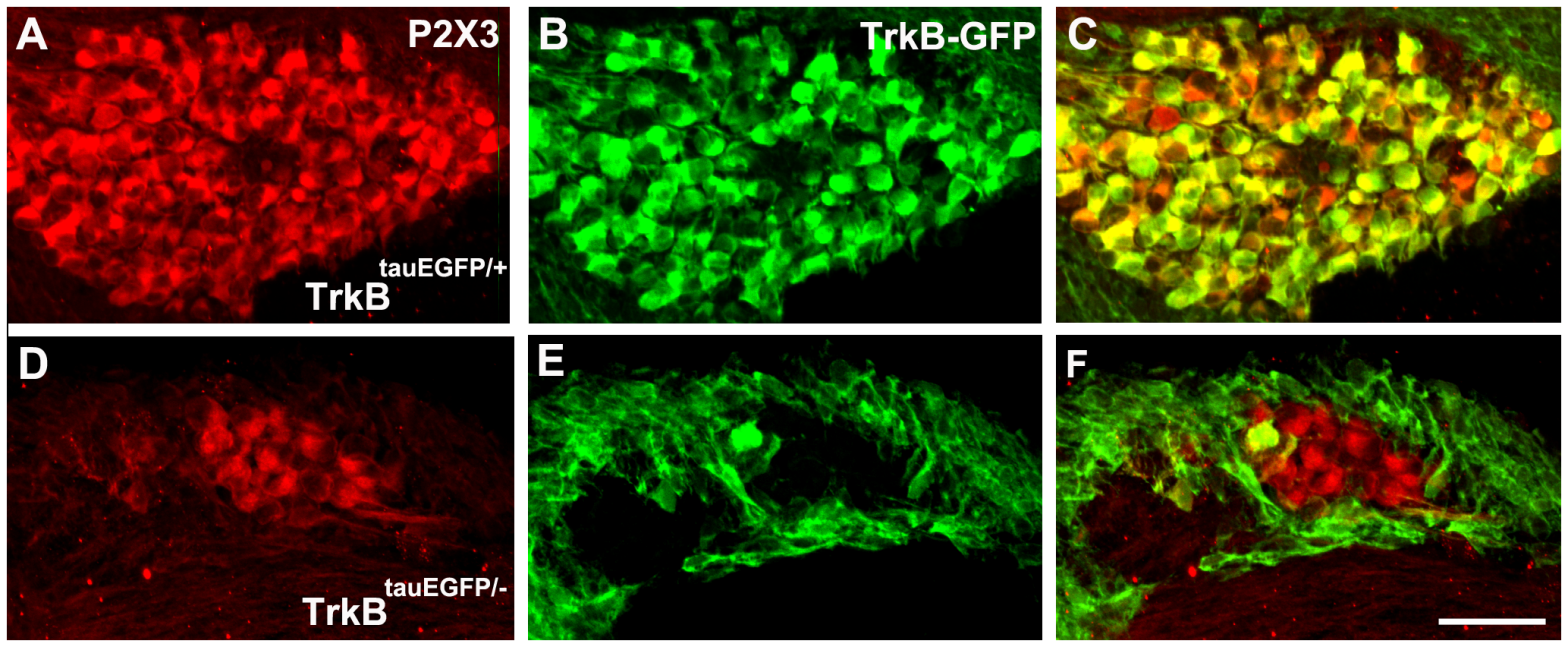
The geniculate neurons that remain in *TrkB^−/−^* mice do not express TrkB. Anti-P2X3 (red) was used to label most neurons in the geniculate ganglion of E13.5 embryos (A, D) and anti-GFP (green) was used to label TrkB-positive geniculate neurons (B, E). The connective tissue surrounding the ganglion was TrkB-GFP positive in both genotypes. The geniculate ganglion from the TrkB^tauEGFP/−^ mouse is smaller and contains few GFP positive neurons then the geniculate ganglion from the TrkB^tauEGFP/−^ mouse. Merged pictures are shown (C, F). The scale bar = 50 µm and applies to A–F.

The reason why gustatory neurons remain and provide greater innervation to the tongue in *TrkB^−/−^* compared with *Bdnf^−/−^/Ntf4^−/−^* mice is unclear. Because the remaining neurons provide more innervation to the taste bud in *TrkB^−/−^* mice than *Bdnf^−/−^/Ntf4^−/−^* mice, the implication is that these neurons can respond to either BDNF or NT-4. The TrkB receptor has a full-length signaling form as well as truncated forms that may or may not signal [28], [29]. The *TrkB^−/−^* mice used in this study still express truncated forms of TrkB [29], [30]. One possibility is that the innervation observed in *TrkB^−/−^* mice is maintained by a truncated form of the TrkB receptor that binds BDNF and/or NT-4 [31]. Alternatively, the subpopulation of neurons that remain in the absence of the full-length TrkB receptor may lack all forms of TrkB and be maintained by an alternate mechanism. To determine whether the remaining neurons in *TrkB^−/−^* mice express any form of the TrkB receptor, *TrkB^tauEGFP/+^* mice were bred with *TrkB^+/−^* mice to obtain *TrkB^tauEGFP/−^* littermates. In *TrkB^tauEGFP/+^* mice, GFP is expressed in all cells that would normally express either truncated or full-length forms of the TrkB receptor [14]. Because *TrkB^−/−^* mice at E13.5 have already lost most of the geniculate neurons, we examined *TrkB^−/−^* mice at this age to determine whether the remaining neurons in *TrkB^−/−^* mice express TrkB. Geniculate neurons were double-stained with anti-P2X3 (red) and anti-GFP (green) (Figure 8). In the wild-type mice, 94±1% of the geniculate neurons that expressed P2X3 also expressed TrkB. In contrast, only 11±2% of the P2X3-positive geniculate neurons expressed GFP in the *TrkB^tauEGFP/−^* mice. This result indicates that most of the neurons that remain in TrkB mutants belong to the 6% of neurons that are negative for all forms of the TrkB receptor. Together with our other findings, this result suggests that there exists a small population of TrkB-independent neurons in the geniculate ganglion that do not express TrkB, but need BDNF and/or NT-4 to regulate innervation patterns and support post-natal taste bud development.

## Discussion

The neurotrophins BDNF and NT-4 play fundamental roles in the regulation of gustatory neuron survival and the ability of gustatory neurons to innervate their peripheral targets during development [1], [3], [5]–[8], [12], [13], [15], [17], [32]–[36]. However, the roles of the TrkB and p75 receptors are less clear. Here, we compared taste neuron survival, taste bud development, and target innervation in *Bdnf^−/−^/Ntf4^−/−^* and *TrkB^−/−^* mice to determine the extent to which BDNF and NT-4 act via TrkB to regulate taste development. Our data show that *Bdnf^−/−^/Ntf4^−/−^* and *TrkB^−/−^* mice lose a similar number of geniculate ganglion neurons by E13.5. In addition, *Bdnf^−/−^/Ntf4^−/−^* and *TrkB* ^−/−^ mice also lose most innervation to the tongue as well as most of their taste buds. These findings indicate that BDNF and NT-4 act primarily via TrkB to regulate the survival of taste neurons.

Subtle differences between the *Bdnf^−/−^/Ntf4^−/−^* and *TrkB^−/−^* mice provide insight into the possible role of other neurotrophins and receptors in the regulation of taste development. For example, the loss of geniculate neurons was slightly delayed in *Bdnf^−/−^/Ntf4^−/−^* compared with *TrkB^−/−^* mice at E12.5. One possible explanation is that other neurotrophins act via the TrkB receptor as survival factors at this stage. The most likely candidate is neurotrophin-3 (NT-3), which acts via TrkB to support DRG neuron survival [37]. NT-3 is robustly expressed in the lingual epithelium, the fungiform papillae core, and the tongue mesenchyme [8], [32], [38]–[40], and has been shown to regulate gustatory neuron functional development [32]. Approximately one-third of the geniculate and nodose-petrosal ganglion depend on NT-3 for survival [41]–[43], while only 11% of geniculate neurons depend on TrkC [44], which indicates that NT3 might also function through TrkB to support geniculate neuron survival. Another possibility is that in the absence of TrkB, BDNF binds p75 to increase cell death of geniculate neurons; BDNF causes cell death of sympathetic neurons by binding p75 [45], [46]. Lastly, the delayed neuron loss in *Bdnf^−/−^/Ntf4* ^−/−^ mice compared with *TrkB* ^−/−^ mice may also be ligand independent. Several growth factors receptors, including TrkB, can function at low levels independent from ligand binding [47]–[49]. Regardless of the underlying mechanism, TrkB signaling in the absence of BDNF and NT-4 can maintain the number of geniculate neurons only temporarily: by E13.5, the effects of TrkB removal and TrkB ligand removal on the number of geniculate neurons are similar.

We observed that more taste buds are present in *TrkB^−/−^* compared with *Bdnf^−/−^/Ntf4^−/−^* mice on the day of birth. This finding is consistent with previous reports that *TrkB^−/−^* mice [11], but not *Bdnf^−/−^/Ntf4^−/−^* mice [13], retain a substantial number of taste buds. A previous examination of *TrkB* ^−/−^ mice claimed that these mice lose most of their geniculate neurons and have no remaining innervation to the tongue [11]. We quantified innervation to the tongue to re-examine whether taste buds survive to birth in the absence of innervation in *TrkB* ^−/−^ mice and to determine why there were more taste buds in *TrkB* ^−/−^ compared with *Bdnf^−/−^/Ntf4^−/−^* mice. We found that neurons innervated more of the remaining taste buds with larger amounts of innervation in *TrkB^−/−^* mice than in *Bdnf^−/−^/Ntf4^−/−^* mice. We also found significantly more taste fibers in *TrkB^−/−^* compared with *Bdnf^−/−^/Ntf4^−/−^* mice. Based on these experiments, we concluded that the increase in taste bud survival in *TrkB^−/−^* mice compared with *Bdnf^−/−^/Ntf4^−/−^* mice is due to the significant innervation that remains in the tongues of *TrkB^−/−^* mice. How NT4 and BDNF function to maintain more innervation to the taste bud in the absence of TrkB is unclear. However, one obvious explanation is that these BDNF and/or NT4 can function via p75 to support targeting and taste bud innervation.

We observed similar numbers of non-innervated taste buds, which do not occur in wild-type mice, in both mutant genotypes. These taste bud remnants were smaller than normal taste buds. These results demonstrate that some keratin 8-stained profiles do not require innervation to be maintained until birth in mice. Neither *Bdnf^−/−^/Ntf4^−/−^* or *TrkB* ^−/−^ survive much past birth, so it is not possible to track changes in postnatal taste bud numbers in *TrkB^−/−^* compared with *Bdnf^−/−^/Ntf4^−/−^* mice. Taste buds are more dependent on innervation for their survival in postnatal development than they are in adulthood [18], [19]. Therefore, we could predict that uninnervated taste buds would be lost during this time, while innervated taste buds could remain. Alternatively, the presence of taste bud remnants independent of nerve fibers is consistent with mouse studies in which some keratin 8-positive groups of cells remain following nerve sectioning [50]. The non-innervated remnants observed in our study do not appear to have normal taste bud morphology and may or may not differentiate into functional taste cell types. Therefore, the question as to whether taste buds remain in the absence of innervation depends largely on the definition of taste buds.

Our findings showed that the tongues of *TrkB^−/−^* mice retained approximately one-third of the normal number of taste buds compared with wild-type mice, although only one-tenth of the normal number of geniculate neurons remained to innervate the tongue. Although the remaining number of taste buds is large compared with the loss of geniculate neurons, these results are consistent with studies that demonstrate that taste bud loss cannot be predicted from neuron loss [12]. For example, *Ntf3^−/−^* mice lose geniculate neurons [42], [43] but do not lose taste buds [7], and *Ntf4^−/−^* mice lose approximately half of the geniculate neurons but lose only a few taste buds by birth [3], [12]. The reason that taste buds remain despite a significant loss of geniculate neurons is because each taste bud is innervated by multiple geniculate neurons [51]. In *TrkB^−/−^* mice, the 194 (97/side) remaining geniculate neurons are more than sufficient to innervate and maintain the 31±3 remaining taste buds. Because similar numbers of neurons remain in the geniculate ganglion of *Bdnf* ^−/−^/*Ntf4* ^−/−^ and *TrkB* ^−/−^ mice, it follows that the number of neurons available in *Bdnf* ^−/−^/*Ntf4* ^−/−^ mice should be sufficient to maintain 31 taste buds. However they do not; this finding indicates that *Bdnf* ^−/−^/*Ntf4* ^−/−^ mice exhibit a disruption in the target innervation of the remaining afferents, which is consistent with observations in *Bdnf* ^−/−^ mice [5]. Therefore, the failure of these remaining neurons to reach their targets successfully contributes to the loss of taste bud innervation in *Bdnf* ^−/−^/*Ntf4* ^−/−^ mice.

If *TrkB^−/−^* and *Bdnf^−/−^/Ntf4^−/−^* mice lose approximately the same number of geniculate ganglion neurons during development and the reduced innervation in *Bdnf^−/−^/Ntf4^−/−^* mice is due to disrupted targeting, then these findings suggest that target innervation is not disrupted in *TrkB^−/−^* mice. We observed that the small number of geniculate ganglion neurons that remain in *TrkB^−/−^* mice were able to innervate the tongue surface despite lacking all forms of the TrkB receptor. While we did find that a few TrkB-positive geniculate neurons remain in TrkB^−/−^ mice, this number would not be sufficient to support the remaining taste buds. Together these results suggest that TrkB-negative geniculate neurons are responsive to BDNF, NT-4 or both. Other neurotrophins and their receptors may be responsible for the survival of the remaining geniculate neurons [52], [53], such as the NT-3 receptor TrkC and the receptor for the Gdnf family of ligands, Ret [54]–[56]. However, BDNF and NT-4 must somehow be responsible for the ability of these neurons to innervate the lingual epithelium, since innervation is completely disrupted in *Bdnf^−/−^/Ntf4^−/−^* mice. One possible mechanism for this innervation is the p75 receptor, which binds BDNF and NT-4 in addition to other neurotrophins. NT-4 may also function through another Trk receptor. For example, NT-4 may stimulate TrkA in some circumstances [57]. Regardless of the mechanism, our results indicate that there exists a small subpopulation of TrkB-negative geniculate neurons that depend on either BDNF or NT-4 to innervate taste buds.

The presence of a TrkB-negative gustatory subpopulation is intriguing, because it demonstrates that gustatory neuron subpopulations exist in the ganglion and perhaps can be identified based on the expression of factors that regulate taste neuron development. In the developing dorsal root ganglion (DRG), neuron diversity is established through a step-wise program that includes both transcription factors and Trk receptors [58] that define different functional classes or groups of DRG neurons. Prior to the current study, all taste neurons were presumed to depend on TrkB [11]. In the current study, we show that a small TrkB-negative subpopulation exists in the geniculate ganglion and supports taste buds. This TrkB-independent subpopulation may have a specific functional taste role. This result raises the possibility that TrkB-expressing neurons that co-express other growth factor receptors, like Ret [54] or TrkC [55], [59] could also define functional subpopulations. In addition, some gustatory neurons may lose TrkB receptor expression during post-natal development [53], [60] to define another subpopulation of taste neurons. The presence of gustatory neuron subpopulations that are defined by the expression of developmental factors opens several future directions of research.
